# Supplementary material for: Reconfigurable signal modulation in a ferroelectric tunnel field-effect transistor
Source: Nat Commun. 2023 May 3;14:2530. doi: 10.1038/s41467-023-38242-w (PMC10156808; doi:10.1038/s41467-023-38242-w)
Supplement: Supplementary file 1 — Supplementary Information [file 41467_2023_38242_MOESM1_ESM.pdf]

Supplementary Information for

Reconfigurable Signal Modulation in a Ferroelectric Tunnel Field-Effect Transistor

Zhongyunshen Zhu[1]*, Anton E. O. Persson[1], and Lars-Erik Wernersson[1]

[1]Department of Electrical and Information Technology, Lund University, 221 00 Lund, Sweden

* E-mail: zhongyunshen.zhu@eit.lth.se

Content

Supplementary Figures 1-9

Supplementary Note 1

Supplementary References 1-2

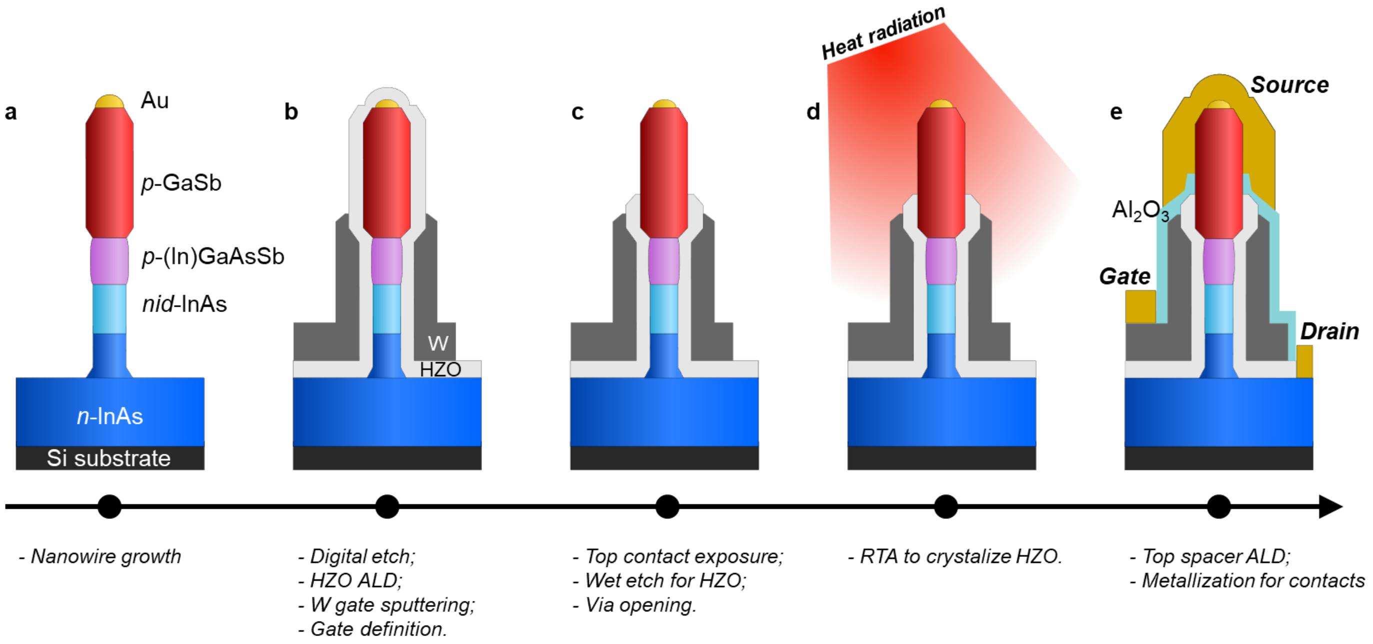

Supplementary Fig. 1. Schematics of the processing flow for ferro-TFET fabrication. a, Nanowire growth by MOVPE. **b**, HZO/W oxide/metal gate-stack deposition. **c**, Top HZO etching for the source contact and opening of gate and drain vias. **d**, HZO was crystalized by RTA at 450 °C for 30 s in a nitrogen ambient. **e**, Top spacer Al_2O_3 deposited by ALD process. The device was finalized by metallization of all contacts.

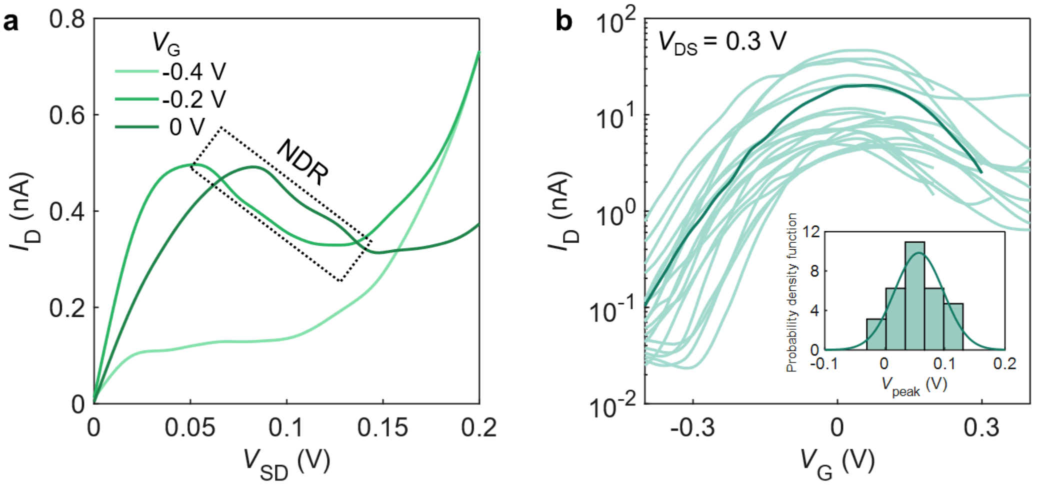

Supplementary Fig. 2. Negative differential resistance (NDR) in ferro-TFETs and device reliability. a, The corresponding negative differential resistance (NDR) from the same ferro-TFET used in this work for reconfigurable signal modulation. The NDR behaviour is obtained by reversing the source and drain bias in the measurement. The result of NDR indicates a high-quality tunnel junction within the device and confirms that band-to-band tunnelling dominates the carrier transport. **b**, Transfer characteristics of 20 devices on the same sample. The inset shows the corresponding V_{peak} distribution, indicating a median V_{peak} sitting at V_G = ~0.05 V and a variation of about ±50 mV. The variation of V_{peak} and I_D among devices is mainly caused by the processing variations but this does not change the conclusions of the work.

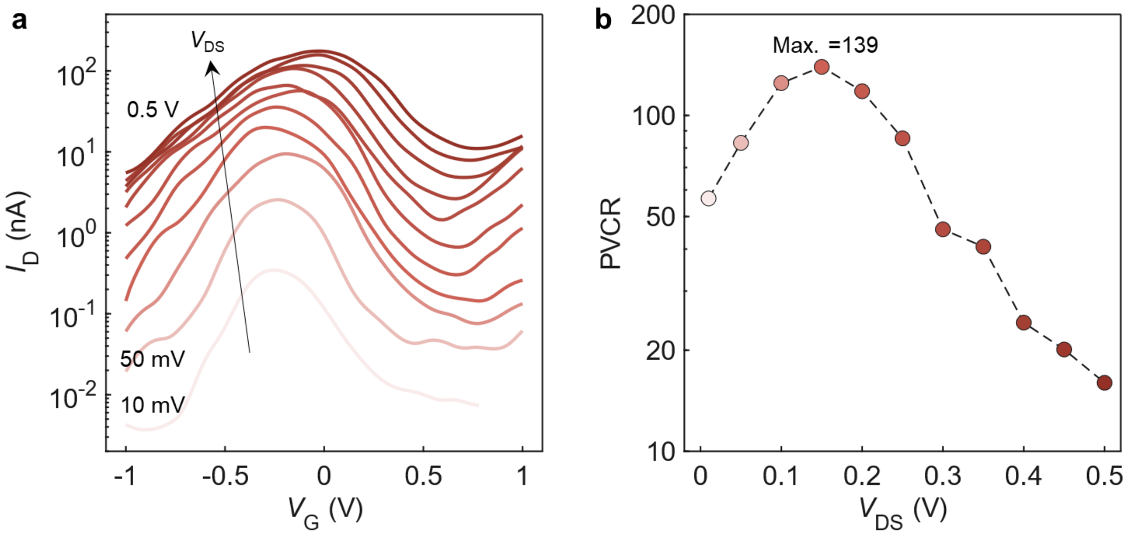

Supplementary Fig. 3. PVCR of NTC in the ferro-TFET in the low-V_{peak} state after ferroelectric switching. a, Transfer characteristic (logarithmic plot) with various V_{DS}. **b**, PVCR as a function of VDS shows that the highest PVCR reaches over 2 orders of magnitude and the value decreases as V_{DS} increases after the maximum PVCR.

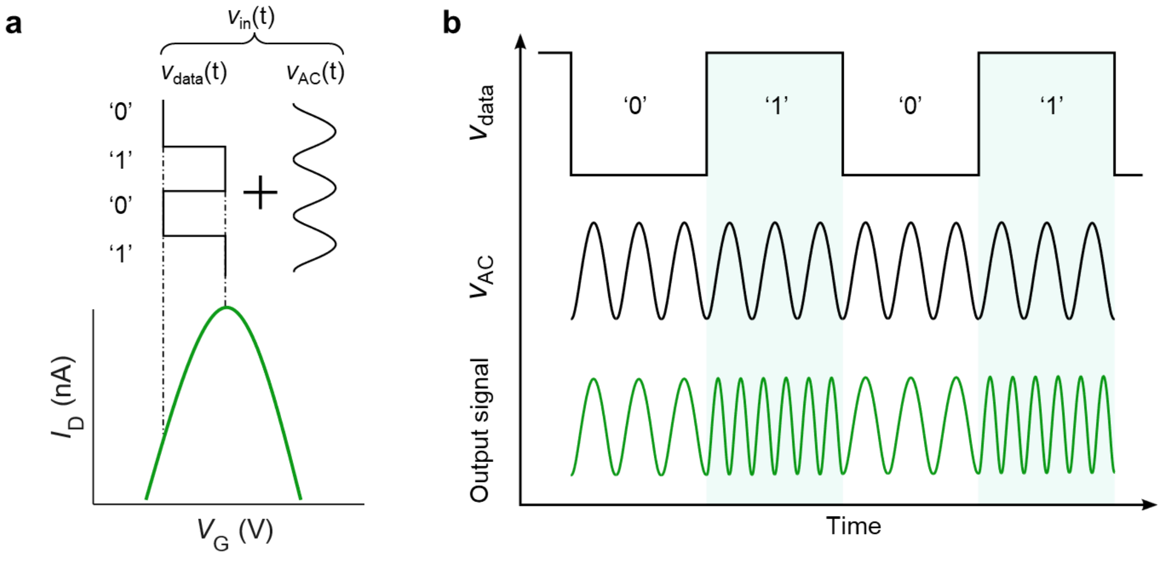

Supplementary Fig. 4. Operating principle of the binary frequency-shift keying (BFSK) in a device with parabolic I_D-V_G. a, Generally, the data is encoded as a periodic DC offset (v_{data}) and is used to shift the input AC signal (v_{AC}) in order to control the frequency modulation. **b**, The input and output waveforms based on the principle described in **a** where v_{data} = '0' for $f_{out} = f_{in}$ while v_{data} = '1' for $f_{out} = 2\times f_{in}$.

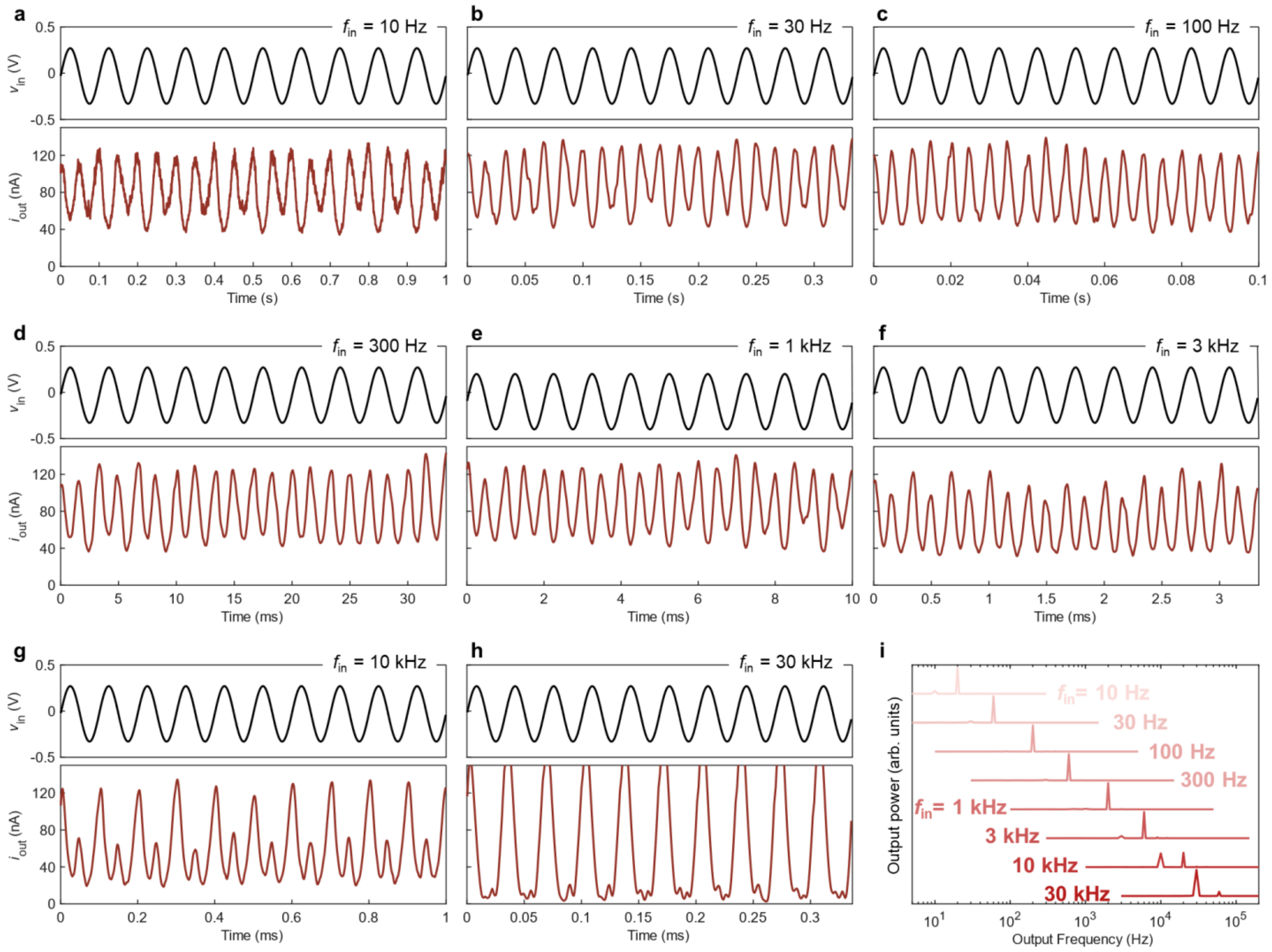

Supplementary Fig. 5. Frequency doubling in the low-V_{peak} state with varying the input frequency. a-h, Time-domain v_{in}-i_{out} characteristics with different input frequencies (f_{in}). When gradually increasing f_{in} up to 3 kHz, the frequency doubling remains undistorted but starts to reduce for f_{in} above 10 kHz. **i**, Power spectra distinctly exhibits $f_{out} = 2f_{in}$ with f_{in} ranging from 10 Hz to 3 kHz. However, when further increasing f_{in} to 10 kHz, the output power at 10 kHz and 20 kHz are almost equal, indicating slight distortion of the frequency doubling. At f_{in} = 30 kHz, the measured f_{out} is identical to f_{in}, showing that no frequency multiplication occurs. Also, it is noticeable that the output current increases significantly. We attribute this to high contribution from the capacitive current (parasitic capacitance) originating from the HZO film between the gate and drain as well as the Al_2O_3 top spacer (10 nm) between the gate and the source in this device structure, which is not optimized for RF operations. The estimated parasitic capacitance between gate and the drain is ~10^5 larger than that of the oxide capacitance. Therefore, by inserting low-permittivity spacers such as hydrogen silsesquioxane (HSQ) or SiO_2 between electrodes, the limited operational frequency may be extended up to 1 GHz. Changes in the nanowire heterostructure, such as modifying the composition of the source material[1] or the doping concentration[2], may further increase the peak current to the benefit of higher frequency operation by negating the parasitic effect.

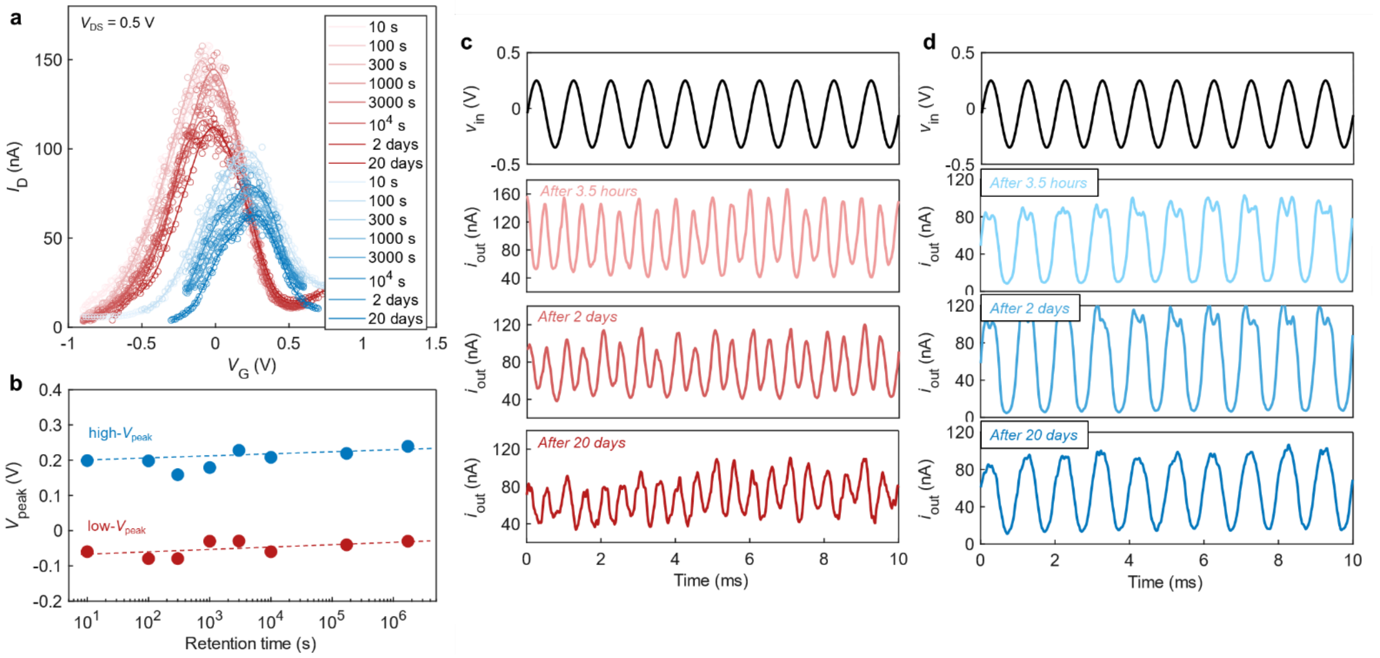

Supplementary Fig. 6. Retention time of reconfigurable NTC property in the ferro-TFET. a, Transfer characteristics of two polarization states with different retention times. **b**, V_{peak} in the two states determined from transfer characteristics measured after a certain retention time. Despite slight fluctuation of V_{peak} between 300 s and 10^4 s, V_{peak} in both states is retaned for a long time $>10^6$ s. **c-d**, The time-domain i_{out} measured when sending an input sinusoidal wave with f_{in} = 1 kHz in the low-V_{peak} (**c**) and the high-V_{peak} (**d**) state after 3.5 hours, 2 days and 20 days, respectively. The result shows that the frequency doubling still operates well 20 days after setting the state.

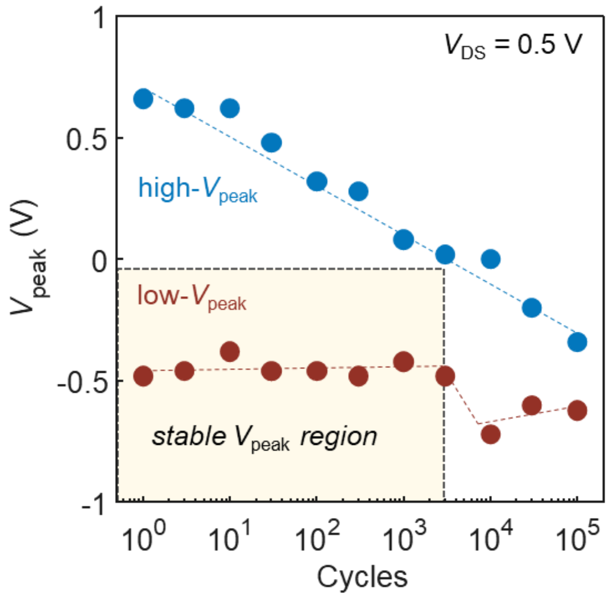

Supplementary Fig. 7. Endurance of reconfigurable NTC property in the ferro-TFET. Here, another device is characterized for a complete dataset from the first pulse cycle. V_{peak} is determined from the transfer curves in two states as a function of number of pulsing cycles. One cycle consists of both a positive (+4 V/250 ns) and a negative (–4 V/250 ns) pulse. The result shows a high endurance over 10^5 cycles. Although the V_{peak} gradually shifts towards more negative voltage while increasing pulsing cycles in the high-V_{peak} state, it remains stable in the low-V_{peak} state during the first 3000 cycles, leading to a stable frequency doubling of i_{out} in the low-V_{peak} state. In the high-V_{peak} state, despite amplitude variation of i_{out} due to V_{peak} shifting, the output frequency characteristic remains identical. After 3000 cycles, the V_{peak} has a slight negative shift but stabilizes again after 10^4 cycles.

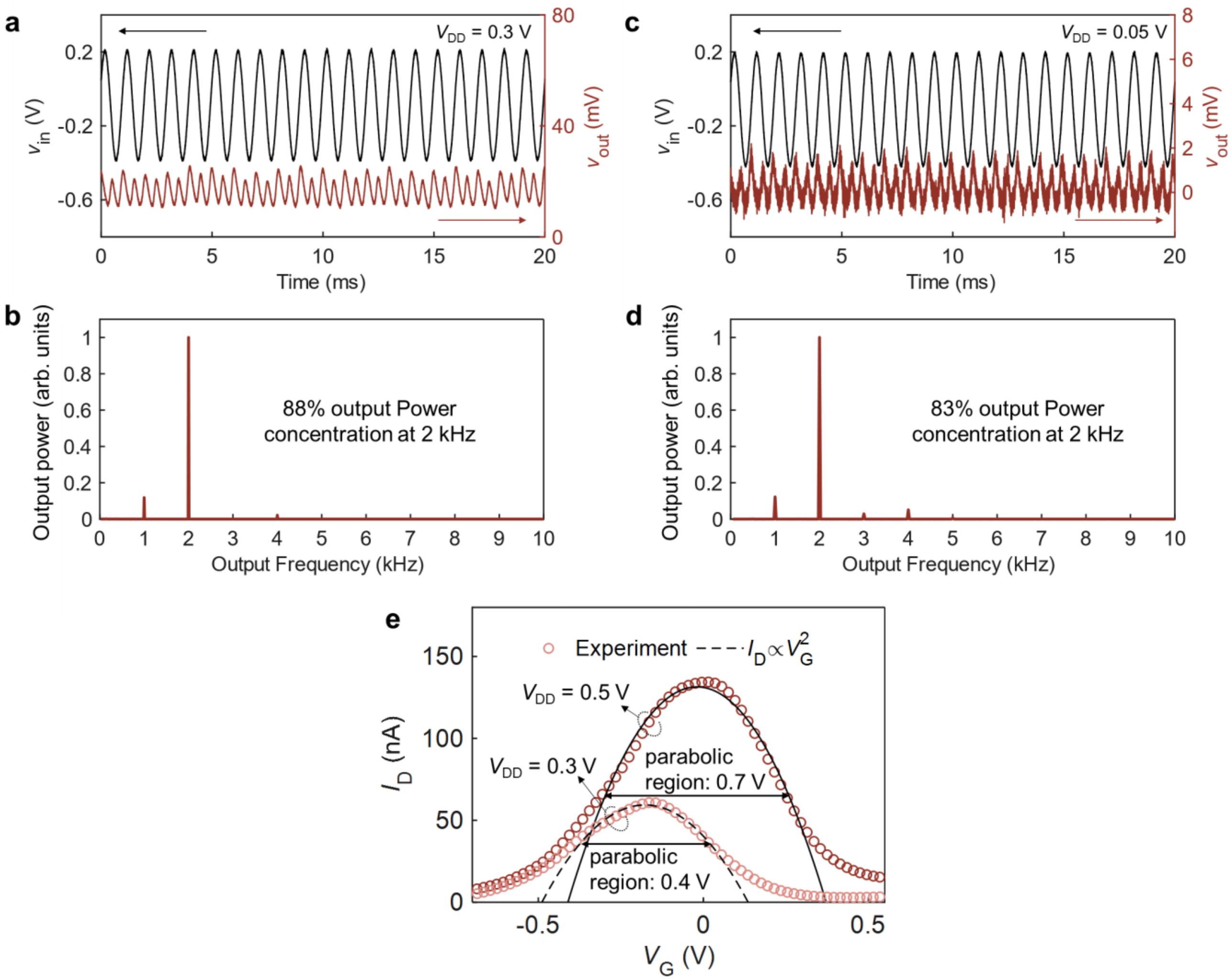

Supplementary Fig. 8. Frequency doubling at low V_{DD}. Time-domain v_{in}-v_{out} characteristics at (**a**) V_{DD} = 0.3 V and (**c**) V_{DD} = 0.05 V with f_{in} = 1 kHz. For a fair comparison, the same amplitude (0.3 V) of the input signal is kept at various V_{DD}. The corresponding power spectrum at V_{DD} = 0.3 V and V_{DD} = 0.05 V is shown in (**b**) and (**d**), respectively. High spectral purity remains when reducing the drive voltage even down to 50 mV. **e**, The I_D-V_G curve in the low-V_{peak} state at V_{DD} of 0.5 V and 0.3 V with corresponding fitting, showing decreased parabolic operation region when decreasing V_{DD}. Notably, the output power at the desired doubled frequency is decreased from 98% to 88% when reducing V_{DD} from 0.5 V to 0.3 V. One reason is a slight output waveform distortion caused by the V_{peak} shift with changing V_{DD}, which generates additional harmonics as the DC-offset of v_{in} is not correspondingly adjusted. Another reason is that the high amplitude part of the input signal is operating beyond the parabolic I_D-V_G region due to a narrower current peak at lower V_{DD}. By tuning the v_{in} DC-offset and reducing its amplitude in accordance with the specific V_{DD}, the concentration of output power at doubled frequency is expected to increase. In a low-power system with some specific application schemes, a small v_{in} may apply to the device. In principle, such a small signal should be detectable with our ferro-TFET and would operate fully within the ideal parabolic I_D-V_G region at an ultra-low V_{DD}.

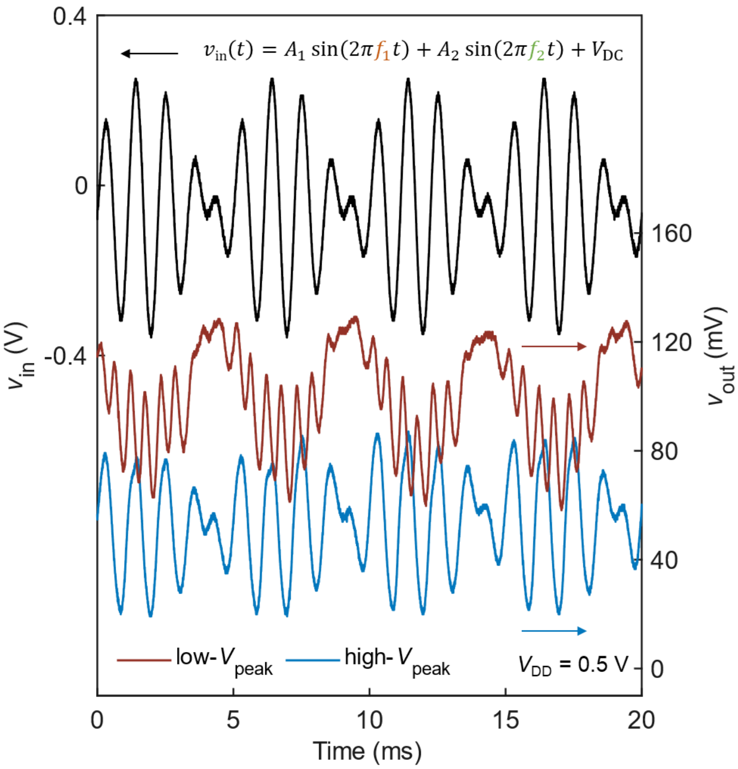

Supplementary Fig. 9. Frequency mixing and transmission in the reconfigurable ferro-TFET. Representative excerpt of the time-domain v_{out} measured when sending a signal (v_{in}) with two sinusoidal waves with f_1 = 1 kHz and f_2 = 800 Hz in the low- and high-V_{peak} state for frequency transmission and mixing, respectively. The crosstalk between two input signals is negligible as the power spectrum (applying fast Fourier transform to entire v_{in}) of v_{in} shows a power of -3 dBm on the desired input signals (800 Hz and 1 kHz) while only < -50 dBm on other harmonics.

Supplementary Note 1: Theoretical derivation of ideal parabolic I_D-V_G curve in frequency mixing

Here, we can assume a parabolic i_{out}-v_{in} (I_D-V_G) curve, which can be expressed as,

$$i_{\mathrm{out}} = -k(v_{\mathrm{in}} - V_{\mathrm{peak}})^2 + I_{\mathrm{peak}} \quad (1)$$

where V_{peak} is the voltage at the current peak which defines the DC offset V_{DC} in v_{in}, I_{peak} the peak current when $v_{\mathrm{in}} = V_{\mathrm{peak}}$, and k the coefficient with the unit of A/V^2. When an input signal consists of two sinusoidal waves with different frequencies (f_1 and f_2), v_{in} can be written as,

$$v_{\mathrm{in}} = A_1 \sin(2\pi f_1 t) + A_2 \sin(2\pi f_2 t) + V_{\mathrm{DC}} \quad (2)$$

where A_1 and A_2 are the voltage amplitudes of the two mixed signals, respectively. When substituting equation (1) into equation (2), we obtain,

$$i_{\mathrm{out}} = I_{\mathrm{peak}} - k\left(A_1^2 \sin^2(2\pi f_1 t) + A_2^2 \sin^2(2\pi f_2 t) + 2A_1 A_2 \sin(2\pi f_1 t) \cdot \sin(2\pi f_2 t)\right)$$

$$= I_{\mathrm{peak}} - kA_1^2 \frac{1 - \cos(2\pi \cdot 2f_1 t)}{2} - kA_2^2 \frac{1 - \cos(2\pi \cdot 2f_2 t)}{2} - kA_1 A_2 \cos[2\pi(f_1 - f_2)t]$$

$$+ kA_1 A_2 \cos[2\pi(f_1 + f_2)t].$$

For simplicity, we here set $A_1 = A_2 = A$ (the same amplitude for two mixed signals) making

$$i_{\mathrm{out}} = \left(I_{\mathrm{peak}} - kA^2\right) + 0.5kA^2 \cos(2\pi \cdot 2f_1 t) + 0.5kA^2 \cos(2\pi \cdot 2f_2 t)$$

$$-kA^2 \cos[2\pi(f_1 - f_2)t] + kA^2 \cos[2\pi(f_1 + f_2)t]. \quad (3)$$

$$v_{\mathrm{out}} = i_{out} \cdot R \quad (4)$$

where v_{out} is the output voltage with a resistor (R) in series with the ferro-TFET as shown in Fig. 4a in the main manuscript. Thus, the signal with frequency of $f_1 - f_2$ or $f_1 + f_2$ has the amplitude twice of that with $2f_1$ or $2f_1$. This agrees well with our frequency spectrum where the intensity ratio between frequency of $f_1 + f_2$ and $2f_1$ (or $2f_2$) is about 2, proving a highly parabolic I_D-V_G existing in the ferro-TFET with NTC.

Supplementary References

1. Krishnaraja A., Svensson J., Memisevic E., Zhu Z., Persson A. R., Lind E., Wallenberg L. R., Wernersson L.-E. Tuning of Source Material for InAs/InGaAsSb/GaSb Application-Specific Vertical Nanowire Tunnel FETs. *ACS Applied Electronic Materials* **2**, 2882-2887 (2020).

2. Memisevic E., Svensson J., Lind E., Wernersson L.-E. Impact of source doping on the performance of vertical InAs/InGaAsSb/GaSb nanowire tunneling field-effect transistors. *Nanotechnology* **29**, 435201 (2018).
